# SUBJECTIVE COGNITIVE DECLINE PHENOTYPES, SOCIAL ENGAGEMENT, AND CARDIOVASCULAR HEALTH

**DOI:** 10.1093/geroni/igad104.2100

**Published:** 2023-12-21

**Authors:** Maritza Dowling, Laurie Theeke

**Affiliations:** George Washington University, Washington, District of COlumbia, United States; The George Washington University, Washington, District of Columbia, United States

## Abstract

Epidemiological studies report subjective cognitive decline (SCD) as a risk factor for incident dementia. The co-occurrence of SCD, chronic conditions, and loneliness may contribute to functional limitations and decline. SCD etiologies are multifaceted, suggesting possible underlying phenotypes defined by clustering factors that characterize decline. Using pooled data (2015-2021) from the Behavioral Risk Factor Surveillance System (BRFSS) we investigated SCD phenotypes based on the six-item self-reported Cognitive Decline Module that measured challenges in daily life due to memory loss and confusion over the prior twelve months. We hypothesized that SCD phenotypes would be associated with socio-demographic, cardiovascular health and social engagement variables. Mixture modeling was used to determine unobserved SCD phenotypes (latent classes) based on item response patterns. Latent class membership was predicted from socio-demographic variables using multinomial logistic regression. The resulting classes predicted a six-item cardiovascular risk index (CVRI) and a measure of aloneness. SCD symptoms were reported by 65,217 (45-80+ years old). Mixture models produced four-latent SCD classes labeled as mild (43%), mild-moderate (23%), moderate (24%), and severe (10%). Mean CVRI scores were significant across classes (p < 0.001) and highest in the severe subgroup (n=6,491) which was more likely to be non-Hispanic Blacks, female, younger (45-64), low-income, non-homeowners, and report depression, and poor/fair health status. Class membership also significantly predicted aloneness. Studies are needed to examine how SCD phenotypes may be used in the design and development of interventions that are more precise to the cluster of modifiable risk factors which could, subsequently, prevent or delay dementia pathogenesis.

